# Characterization of the Specific Binding Between Aptamers and Cytochrome *c* With Pressure‐Assisted Capillary Electrophoresis Frontal Analysis

**DOI:** 10.1002/elps.70018

**Published:** 2025-09-01

**Authors:** Shuanghao Wang, Chunliang Li, Shuangshuang Wang, Huihui Li, David Da Yong Chen

**Affiliations:** ^1^ State Key Laboratory of Microbial Technology, Jiangsu Collaborative Innovation Center of Biomedical Functional Materials, Jiangsu Key Laboratory of New Power Batteries, School of Chemistry and Materials Science Nanjing Normal University Nanjing China; ^2^ Jiangyan High School of Jiangsu Province Taizhou China; ^3^ Department of Chemistry University of British Columbia Vancouver British Columbia Canada

**Keywords:** aptamer | binding constant | capillary electrophoresis frontal analysis | cytochrome *c* | specificity

## Abstract

Cytochrome *c* (cyt *c*) is a heme protein located in the mitochondrial intermembrane space. Because the release of cyt *c* is a highly specific event in apoptotic signaling, it can serve as an apoptosis‐related marker. To date, three frequently used aptamers for cyt *c* (Apt40, Apt61, and Apt76) have been selected and applied in the field of sensing. The response of these aptamers is not clear, partly because of their weak affinity and nonspecific binding inherent to the system. In this study, pressure‐assisted capillary electrophoresis frontal analysis (PACE‐FA) was used to characterize the interactions between the aptamers and cyt *c*, and an electrophoretic mobility‐based correction was introduced to obtain accurate binding constants. A nonlinear curve‐fitting approach was used for evaluating specific binding interactions in the presence of nonspecific binding. Apt76 was found to bind specifically to cyt *c*, exhibiting the highest binding constant (1.53 × 10^6^ M^−1^), and all three aptamers interacted with cyt *c* at 1:1 stoichiometry. Fluorescence titrations were performed to verify the effectiveness of the reference‐free PACE‐FA method. This study demonstrates that specific binding between biomolecules has different characteristics compared to nonspecific binding and that the PACE‐FA method can be widely used in the evaluation of biological macromolecular interactions.

**Abbreviations**
CE‐FA: capillary electrophoresis frontal analysis
cyt *c*
: cytochrome *c*
DNA: deoxyribonucleic acid
HPC: hydroxypropyl cellulose
PACE‐FA: pressure‐assisted capillary electrophoresis frontal analysis
RNA: ribonucleic acid
SELEX: systematic evolution of ligands via exponential enrichment
SPR: surface plasmon resonance

## 1 Introduction

Aptamers are a class of single‐stranded oligonucleotides (ribonucleic acid [RNA] or deoxyribonucleic acid [DNA] mimics) that can recognize specific targets. Aptamers can selectively bind to various targets, such as small molecules, proteins, and even whole cells [1, 2, 3, 4]. Their specific recognition capability and high affinity to their target are comparable to those of antibodies. Since the first report on aptamer‐based molecular recognition in 1990 [5, 6], some aptamers have been widely used as recognition elements in biosensing devices [7, 8, 9], drug delivery [10, 11], diagnostics, and therapeutic agents [12, 13, 14]. Compared to antibodies or enzymes, aptamers exhibit stability over wide temperature and pH ranges and have a longer shelf life. Furthermore, aptamer sequences are not immunogenic, because they are generally not targeted by the immune system.

Aptamers are usually selected by a process called the systematic evolution of ligands via exponential enrichment (SELEX) [5, 15]. Despite the advantages of aptamers as biorecognition elements and their wide‐ranging applications, some aptamer sequences were recently reported to be incapable of binding to their targets [16, 17, 18]. Their functions have been a subject of significant debate, and the accurate evaluation of aptamer binding remains a challenging task. In particular, the specific recognition mechanisms of protein‐targeting aptamers remain unclear. Improper measurements of affinity may result in false‐positive binding results, which affect the reliability of aptamer‐based sensing [19].

It is critical to develop appropriate methods to evaluate the target‐binding affinities of aptamers for their use in bioanalytical applications. Classical binding evaluation methods, including equilibrium dialysis, affinity chromatography, and gel‐based methods, have been used to obtain kinetic and thermodynamic information on binding events and have been discussed comprehensively in the literature [20, 21]. Further, some of the newer methods used in binding assays can be divided into two categories: label‐requiring and label‐free. Label‐requiring methods have one partner of the target–aptamer system fluorescently labeled to quantitatively detect the presence of aptamer–target complexes [22]. For example, assays rely on the conjugation of aptamers with fluorophores or DNA intercalating dyes [23, 24]. Label‐free affinity measurements based on surface plasmon resonance (SPR) validate the binding affinities and kinetic characteristics of targets to surface‐immobilized aptamers by analyzing the changes in the SPR properties of the support [25, 26]. However, the immobilization of aptamers on the supports can inevitably change their secondary structure, which could affect their interactions with specific targets. Native electrospray ionization–mass spectrometry (Native ESI‐MS) is a fast, sensitive, and simple label‐free method for evaluating aptamer–target systems in equilibrium states [27]. Considering that the transition of the material from the solution phase to gas phase in the ionization process may cause the structural deformation of the aptamer–target complex, the ESI‐MS‐based method is not suitable for evaluating the quantitative affinity of weakly bound aptamer–target systems [28].

Capillary electrophoresis (CE) could separate the sample components based on the differences in their electrophoretic mobility and distribution behavior after the sample is injected into a capillary [29]. CE measurements can be automated and yield a greater range of qualitative and quantitative information for each analyte in free solution, provided that appropriate experimental controls are used before sample injection. Given the advantages of low sample consumption and high sensitivity, CE also offers aptamer–target binding characterization in free solutions without requiring immobilization strategies [30]. Affinity capillary electrophoresis (ACE) is commonly used to characterize aptamer–target interactions [31]. ACE‐based SELEX protocols (denoted as CE‐SELEX) can select high‐affinity aptamers, and the duration of the SELEX process can be shortened to a few days [32]. Capillary electrophoresis frontal analysis (CE‐FA) employs a relatively large volume (nanoliter level) of a pre‐equilibrated sample that is injected into a capillary filled with a background electrolyte (BGE). The sample consists of the target, ligand, and BGE. When a separation voltage is applied, the components in the mixture migrate with different mobilities, and the unbound ligand molecules generate a plateau [33]. The height of the plateau, which is proportional to the free ligand concentration in the sample, can be used to quantify target–ligand interactions and obtain affinity constants and reaction stoichiometry. CE‐FA is widely used to study target–ligand interactions because of the simplified sample preparation protocol, easy manipulation, and high separation efficiency [34]. Recently, an improved CE‐FA variant, pressure‐assisted CE‐FA (PACE‐FA), was established [35, 36]. In this process, an appropriate level of pressure is applied, along with the separation voltage, to significantly decrease the analysis time without compromising accuracy. The PACE‐FA method has been used to study interactions between cyclodextrin–drug, protein–ligand, DNA–drug, and other applications [37, 38].

Cytochrome *c* (cyt *c*) is a heme protein found in the intermembrane space of mitochondria. In addition to acting as an electron transporter in biological processes such as ATP synthesis and protein dephosphorylation, cyt *c* plays an important role in cell apoptosis [39, 40]. Fluorescence experiments have indicated that once the apoptosis is activated, cyt *c* is released into the cytosol, where it binds to Apaf‐1 protein to form an apoptosome complex, leading to irreversible apoptosis [41, 42]. cyt *c* not only serves as an apoptotic marker but can also be used to develop apoptosis‐based targeted therapeutic strategies [43]. As a specific target, cyt *c* can be used to modulate pathways and selectively induce apoptosis in tumor cells [44, 45]. Since the first 61‐mer aptamer for cyt *c*, Apt61 (dissociation constant *K*_d_ ∼ 4.6 µM), was screened and reported in 2002 [46]. Two other aptamers (Apt40 and Apt76) were identified [47, 48]. These aptamers are used as recognition elements to detect cyt *c* released from apoptotic cells. However, a simultaneous detection assay based on aptamer‐modified monolithic capillary chromatography indicated a lack of specific binding of Apt61 [49]. Apt61 was found to bind to both cyt *c* and thrombin in real blood samples. According to a previous study based on thickness shear mode acoustics and electrochemical methods, although Apt 76 has a relatively high affinity for cyt *c*, the solution conditions, including the buffer type and ionic strength, could affect the binding of Apt76 to cyt *c* [2].

In this study, we used the PACE‐FA method to comprehensively analyze the interactions between the three different aptamers (Apt40, Apt61, and Apt76) and their target protein, cyt *c*. By optimizing the detection conditions, a stable platform signal of cyt *c* was obtained in the electrogram, and the accuracy of the PACE‐FA results was improved through mobility‐based corrections. Furthermore, using a nonlinear curve‐fitting equation, specific binding constants and stoichiometries were determined in the presence of nonspecific binding, eliminating the need for reference compounds. To verify the obtained binding results, fluorescence titration experiments were performed to compare the characteristics of the cyt *c*–aptamer interactions.

## 2 Experimental Section

### 2.1 Materials

Three aptamers (Apt40: 5′‐CCGTGTCTGGGGCCGACCGGCGCATTGGGTACGTTGTTGC‐3′, Apt61: 5′‐AGTGTGAAATATCTAAACTAAATGTGGAGGGTGGGACGGGAAGAAGTTTATTTTTCACACT‐3′, and Apt76: 5′‐ATCGATAAGCTTCCAGAG CCGTGTCTGGGGCCGACCGGCGCATTGGGTACGTTGTTGCCGTAGAATTCCTGCAGCC‐3′) were synthesized by Sangon Biotechnology Co. Ltd. (China) and purified by HPLC. cyt *c* (Equine heart, ≥95%) was purchased from Sigma‐Aldrich (China). Hydroxypropyl cellulose (HPC), ammonium acetate (NH_4_OAc), HCl, NH_3_·H_2_O, and CH_3_OH were of HPLC grade and purchased from Wanqing Chemical Glassware & Instrument Co. Ltd. (China). All solutions were filtered with a 0.22 µm pore‐size filter membrane (ANPEL Laboratory Technologies, China).

### 2.2 Pressure‐Assisted Capillary Electrophoresis Frontal Analysis

PACE‐FA experiments were performed using a PA800 Plus CE system (Sciex, Framingham, MA, USA) equipped with a photodiode array detector. A neutrally coated HPC capillary (50 µm inner diameter, total length of 60 cm, effective length of 50 cm) was used. The capillary was rinsed at 40 psi (275.8 kPa) with CH_3_OH (5 min), H_2_O (5 min), and 0.1 M NaOH solution (10 min), H_2_O (5 min), 5% (w/w) HPC water solution (50 min) sequentially, and an air‐drying step (20 min). After the rinsing and drying steps, the coated capillary was baked in a chromatographic oven under with a temperature program (heating from 65°C to 140°C at a rate of 5°C/min, then 140°C for 120 min) with N_2_ at 60 psi (413.7 kPa). Before use, the capillary was rinsed with BGE (10 mM NH_4_OAc, pH 6.8) at 10 psi (68.9 kPa) for 30 min. The cyt *c* solution was diluted to a final concentration of 6.0 µM and mixed with the aptamers (0–8.0 µM) in 10 mM NH_4_OAc (pH 6.8) in each experiment. The absorbances of the solutions were measured at 420 nm. The capillary cartridge temperature was set to 25°C. Each sample was tested in triplicate. Origin 8.5 was employed for software data processing.

### 2.3 Fluorescence Spectroscopy

Fluorescence titrations were performed using a Hitachi F‐7100 fluorescence spectrophotometer (Hitachi, Japan) in the wavelength range of 280–480 nm. The excitation wavelength was 262 nm, and the scanning speed was set to 60 nm/min. For each titration experiment, 400 µL of a 2.0 µM cyt *c* solution in 10 mM NH_4_OAc (pH 6.8) was prepared in a 0.1 cm path length cuvette. It was then mixed with the aptamers (2.0–14.0 µM) and incubated for 30 min to ensure equilibration. A spectrum based on an average of three replicates was obtained for each sample. Origin 8.5 was used for data processing.

## 3 Theory

### 3.1 Binding Analysis Using PACE‐FA

Aptamer binding to a target protein can be described using the following equilibrium [50]:

$$A+nC=AC_{n}$$

(1) $$K_{a}=\frac{\left[AC_{n}\right]}{\left[A\right]{\left[C\right]}^{n}}$$

The binding constant, *K*_a_ is given by Equation (1), where [*A*], [*C*], and [*AC_n_*] are the concentrations of the unbound aptamer, unbound cyt *c*, and the complex of the two at equilibrium, respectively.

For the CE‐FA binding assay, samples were prepared to ensure a constant concentration of the aptamer molecules and variable cyt *c* concentration. As the cyt *c* concentration increased, the number of cyt *c* molecules bound to each aptamer molecule increased. The average binding ratio, *r*, was calculated using Equation (2) and used to determine binding constant, *K*_a_, and binding stoichiometry, *n* [51, 52]:

(2) $$r=\frac{{\left[C\right]}_{b}}{{\left[A\right]}_{t}}=\frac{nK_{a}\left[C\right]}{1+K_{a}\left[C\right]}$$

where [*C*]_b_ and [*A*]_t_ represent the bound cyt *c* and total aptamer concentrations, respectively. For a target molecule with multiple binding sites, both the binding constant (*K*_a_) and *n* can be obtained by the nonlinear regression of the binding isotherm data (*r* vs. [*C*] plot). This established CE‐FA measurement is limited to systems in which the target–ligand complex has a mobility equal to the mobility of one of the interacting species. Therefore, the applicability of CE‐FA in real‐life applications typically results in significant errors [53]. The difference in mobility of species (cyt *c* and its complex with the aptamer) affects the measured free cyt *c* concentration; the binding results determined will be inaccurate. Instead of the [*C*] determined from the calibration curve, the mobility‐corrected free cyt *c* concentration [*C*]_f_ was used to represent the actual concentration of free cyt *c* at equilibrium:

(3) $${\left[C\right]}_{f}=\frac{\mu_{\mathrm{ep},AC_{n}}-\mu_{\mathrm{ep},A}}{\mu_{\mathrm{ep},AC_{n}}-\mu_{\mathrm{ep},C}}{\left[C\right]}_{t}-\frac{\mu_{\mathrm{ep},C}-\mu_{\mathrm{ep},A}}{\mu_{\mathrm{ep},AC_{n}}-\mu_{\mathrm{ep},C}}\left[C\right]$$

where [*C*]_t_ represents the total cyt *c* concentration in the mixture. The effective mobilities of the aptamer and cyt *c* and their complexes (*µ*_ep,_ *_A_*, *µ*_ep,_ *_C_*, and $\mu_{\mathrm{ep},AC_{n}}$, respectively) can be measured in separate experiments.

In binding studies, specific bindings are usually accompanied by nonspecific binding. In this study, a method was established to distinguish between specific and nonspecific binding. We expanded Equation (2) by considering nonspecific binding, without the need for reference compounds, and propose an equation that includes both nonspecific and specific binding [54]:

(4) $$r=\frac{nK_{a}{\left[C\right]}_{f}}{1+K_{a}{\left[C\right]}_{f}}+\lambda {\left[C\right]}_{f}$$

Equation (4) consists of two parts: The first part, $\frac{nK_{a}{\left[C\right]}_{f}}{1+K_{a}{\left[C\right]}_{f}}$, represents specific binding, and the second part, *λ*[*C*]_f_, represents nonspecific binding. A factor, *λ*, is introduced, where the portion of nonspecific binding is proportional to the aptamer concentration.

### 3.2 Binding Analysis Using Fluorescence Spectroscopy

For a 1:1 noncovalent binding system in an equilibrated solution, binding constant *K*_a_ can be quantitatively determined using the Benesi–Hildebrand equation [55], which can be expressed as

(5) $$\frac{1}{\left(F_{0}\;-\;F\right)}=\frac{1}{F_{0}}+\frac{1}{K_{a}F_{0}C_{\mathrm{apt}}}$$

where *F*_0_ and *F* denote the fluorescence intensities of cyt *c* in the absence and presence of the aptamer, respectively. *C*_apt_ denotes the concentration of the aptamer added.

## 4 Results and Discussion

### 4.1 Optimization of the PACE‐FA Conditions

The binding constant (*K*_a_) and binding stoichiometry (*n*) of aptamer–target complexes are key parameters that represent the binding interactions between them. In this study, PACE‐FA measurements were performed to determine the binding constants corresponding to the binding of all three aptamers (Apt40, Apt61, and Apt76) to cyt *c*. On the basis of the satisfactory plateau‐shaped peak shape and signal‐to‐noise ratio of cyt *c* binding, an ammonium acetate solution was selected as the BGE for the PACE‐FA experiments. In an ammonium acetate solution (pH 6.8), cyt *c* is positively charged, whereas the aptamers are negatively charged. In a capillary with the BGE, they would have significantly different electrophoretic mobilities. To reduce the adsorption of the positively charged proteins to the inner capillary wall, a neutrally coated HPC capillary was used for CE [56]. In PACE‐FA, a plateau‐shaped signal was obtained in response to the cyt *c* molecules, and the height of the plateau correlated with the concentration of the unbound protein in the equilibrated system.

**FIGURE 1:**
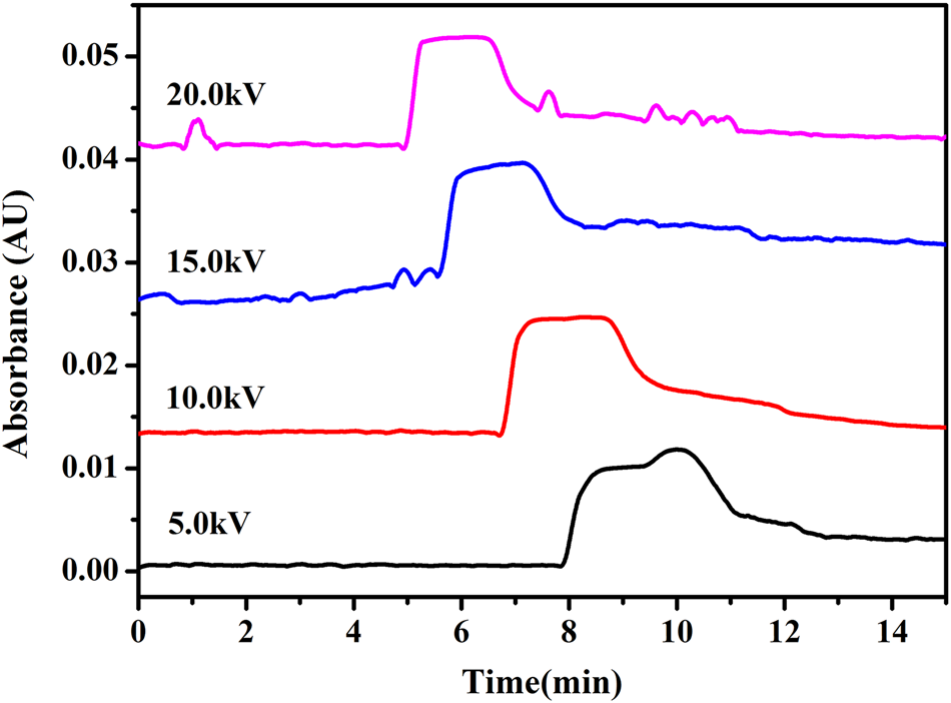
PACE‐FA electropherograms of an 8.0 µM cyt *c* solution in 10 mM NH_4_OAc at different separation voltages. Injection time: 90 s, injection pressure: 1.0 psi (6.9 kPa), external pressure: 2.0 psi (13.8 kPa).

To comprehensively understand the factors that could potentially affect the plateau signal of the analyte in PACE‐FA, we examined the effects of the injection pressure, injection time, separation voltage, and external pressure. Because the charged solutes migrate faster under higher voltages, less time is required for the sample plug to pass by the detector. Thus, as shown in Figure 1, the time‐domain plateau became narrower as the magnitude of the applied voltage was increased. A nearly rectangular plateau was obtained within the tested voltage range of 15–20 kV. The plateau profile did not change significantly until the voltage was decreased to 5–10 kV. It was found that the lowest standard deviation (about 1.8%) in plateau height of the platform was obtained from the CE‐FA results when the separation voltage of 20 kV for the three duplicate runs. Considering the repeatability of the results, 20 kV was selected as the optimal separation voltage for further investigations.

We also studied the possible effects of the external hydrodynamic pressure. The use of external pressure during the CE‐FA process not only significantly reduces the analysis time but also helps maintain a level of accuracy that is comparable to that of the conventional CE‐FA method [35]. Figure S1 compares the electropherograms obtained through PACE‐FA at different pressures in the range of 0.5–2.0 psi (3.4–13.8 kPa). As the pressure increased, the solute molecules migrated at a significantly faster rate. Consequently, the time‐domain plateau became narrower, and the plateau height increased with increasing pressure. If the plateau shape is maintained, the analysis speed can be increased further by increasing the pressure in the PACE‐FA system. Therefore, 2.0 psi (13.8 kPa) was selected as the external pressure in the PACE‐FA process.

Having optimized the external pressure, the effects of the injection pressure and injection time were studied. For this, PACE‐FA experiments were carried out using an 8.0 µM cyt *c* solution in 10 mM NH_4_OAc (pH 6.8), and the injection pressure was varied in the range of 0.5–2.0 psi (3.4–13.8 kPa). As shown in Figure S2, when the injection pressure was 0.5 psi (3.4 kPa), a sharp peak appeared instead of a plateau in the PACE‐FA electropherogram of the cyt *c* solution. When the injection pressure was increased to 1.0 psi (6.9 kPa), a plateau peak appeared. Considering the peak shape, repeatability of the results, and the small sample quantity, an injection pressure of 1.0 psi (6.9 kPa) was chosen as the optimal condition. Similarly, the injection time (90–180 s) was adjusted to maintain a smooth peak shape for the analyte flow in the capillary (Figure S3). On the basis of the optimized results, 90 s was selected as the injection time for subsequent PACE‐FA experiments.

### 4.2 Quantitative Analysis of the Interactions Between cyt *c* and the Three Aptamers Using PACE‐FA

**FIGURE 2:**
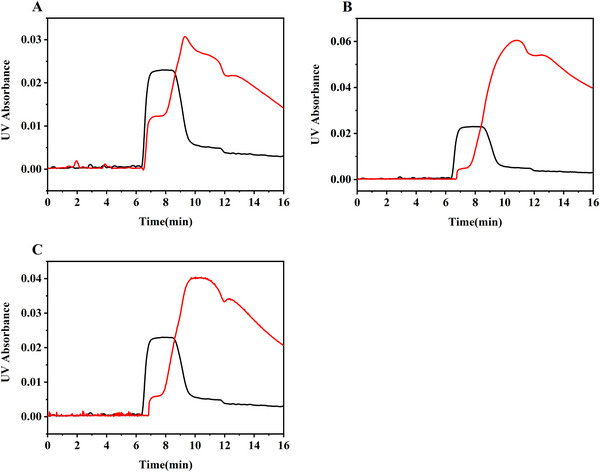
PACE‐FA electropherograms obtained using a 6.0 µmol/L cyt *c* solution as the standard (black) and a pre‐equilibrated mixture of 6.0 µmol/L cyt *c* and 3.5 µmol/L aptamer (red) as the experimental group: (A) Apt40, (B) Apt61, and (C) Apt76. BGE: NH_4_OAc, pH 6.8. Injection time: 90 s. Injection pressure: 1.0 psi (6.9 kPa). Separation voltage: 20 kV. External pressure: 2.0 psi (13.8 kPa).

**FIGURE 3:**
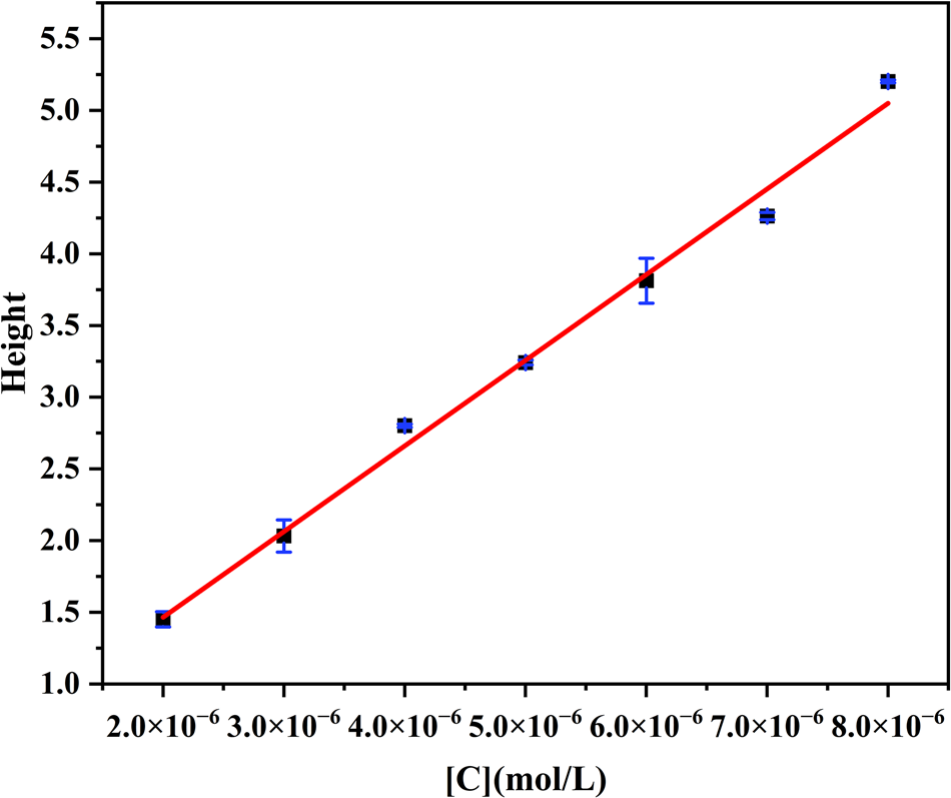
Standard calibration curve of cyt *c* obtained by the PACE‐FA method. BGE: NH_4_OAc, pH 6.8. Injection time: 90 s. Injection pressure: 1.0 psi (6.9 kPa). Separation voltage: 20 kV. External pressure: 2.0 psi (13.8 kPa).

The binding constants of the cyt *c*–aptamer pairs were determined using the PACE‐FA method. Compared to the ACE methods, PACE‐FA quantifies by analyzing the plateau height formed at the leading edge of the sample zone, exhibiting higher tolerance for peak broadening or tailing. This is a key advantage demonstrated by the application of this method in the cyt *c*–aptamer interactions. Figure 2 shows representative electropherograms obtained before and after aptamer addition to a cyt *c* solution. The results indicate that the positively charged cyt *c* molecule migrated first, followed by the cyt *c*–aptamer complex formed by the interaction of the two. During solute separation using the PACE‐FA method, a plateau‐shaped signal of the cyt *c* molecules migrating out of the sample plug of the aptamer and the cyt *c*–aptamer complex is obtained. The CE‐FA curves have similar plateaus on the left side, generated by the free cyt *c* from the mixture. A significant amount of complex still existed in the plug at the end of the run, giving rise to a peak on the right side. Due to the amount of complex formed in the preequilibrated sample, the concentration of free cyt *c* decreases, and the height of the free cyt *c* plateau also decreases compared to the plateau obtained for the sample containing cyt *c* only. The height of the plateau in the electropherogram is directly related to the concentration of the unbound cyt *c* molecules in the equilibrated cyt *c*/aptamer mixture, and the unbound cyt *c* concentration can be determined using a standard calibration curve. Figure 3 shows the standard calibration curve obtained for cyt *c* in the concentration range of 2.0–8.0 µM.

**FIGURE 4:**
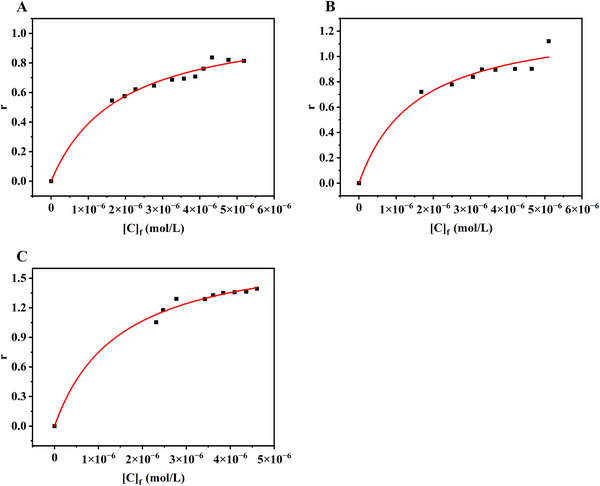
Nonlinear regression curves of the mobility‐corrected PACE‐FA data: (A) cyt *c*–Apt40, (B) cyt *c*–Apt61, and (C) cyt *c*–Apt76.

To obtain accurate binding constants of the cyt *c*–aptamer complexes, the conventional CE‐FA data‐processing method imposes specific requirements on the mobilities of the binding pairs. It has been suggested that the main discrepancy in the measurement of the free analyte concentration in a target–ligand complex mixture originates from the difference in mobility of species. In our previous study, we formulated a new equation to effectively correct the systematic error caused by the mobility differences of various species of binding pairs that did not fulfill the requirement [53]. Additionally, applying a controlled hydrodynamic pressure (≤2 psi) during the CE‐FA process helped reduce the time of binding analysis while maintaining measurement accuracy. All the datapoints plotted in Figure 4 were processed using a mobility‐based correction, and Table 1 summarizes the binding constants (*K*_a_ values) obtained after the nonlinear regression analyses of the obtained data. As the overestimation of the unbound cyt *c* fraction in the analytical mixture due to the dissociation of the cyt *c*–aptamer complex could not be prevented, we introduced mobility terms into Equation (3) to determine the actual concentration of unbound cyt *c* at equilibrium. The PACE‐FA results revealed that the three aptamer molecules bind to cyt *c* with similar binding constants of the order of 10^5^ M^−1^. The *K*_a_ value of the cyt *c*–Apt61 complex is comparable to that obtained previously using absorption spectroscopy [46]. The results revealed that cyt *c* binds to Apt40 and Apt61 at an approximate ratio of 1:1, whereas it binds to Apt76 at a much higher ratio (cyt *c*:aptamer ≈ 2:1).

**TABLE 1:** Summary of the binding parameters for the interactions of cyt *c* and the three aptamers (Apt76, Apt40, and Apt61) using different methods.

Aptamer	PACE‐FA									Fluorescence titration	
	Raw data			Mobility correction			Specific binding			*K*_a_ (M^−1^)	*R* ^2^
	*K*_a_ (M^−1^)	*n*	*R* ^2^	*K*_a_ (M^−1^)	*n*	*R* ^2^	*K*_a_ (M^−1^)	*n*	*λ* (M^−1^)		
Apt40	2.01 × 10^6^	1.06	0.96	5.45 × 10^5^	1.10	0.98	8.40 × 10^5^	0.84	2.83 × 10^4^	1.51 × 10^5^	0.99
Apt61	1.06 × 10^6^	1.47	0.96	6.43 × 10^5^	1.30	0.96	7.12 × 10^5^	1.22	7.11 × 10^3^	6.25 × 10^5^	0.99
Apt76	1.65 × 10^6^	2.05	0.99	6.76 × 10^5^	1.85	0.99	1.53 × 10^6^	1.25	6.90 × 10^4^	8.68 × 10^5^	0.99

Abbreviation: PACE‐FA, pressure‐assisted capillary electrophoresis frontal analysis.

### 4.3 Specific Binding Ability of the Three cyt *c* Aptamers

Specific binding is crucial for the aptamer‐based recognition of target molecules. However, in a binding interaction, specific binding is usually accompanied by nonspecific binding, which is often mediated by electrostatic interactions. Notably, the ligand preferentially binds to a specific binding site of the target. However, specific binding sites are extremely limited. As the ligand concentration increases, the specific binding sites are almost completely occupied, and the excess ligand molecules bind nonspecifically to the target molecule, thereby extending the binding curve. It is, therefore, imperative to use an effective method that can characterize both specific and nonspecific binding for extracting the information related to specific binding.

**FIGURE 5:**
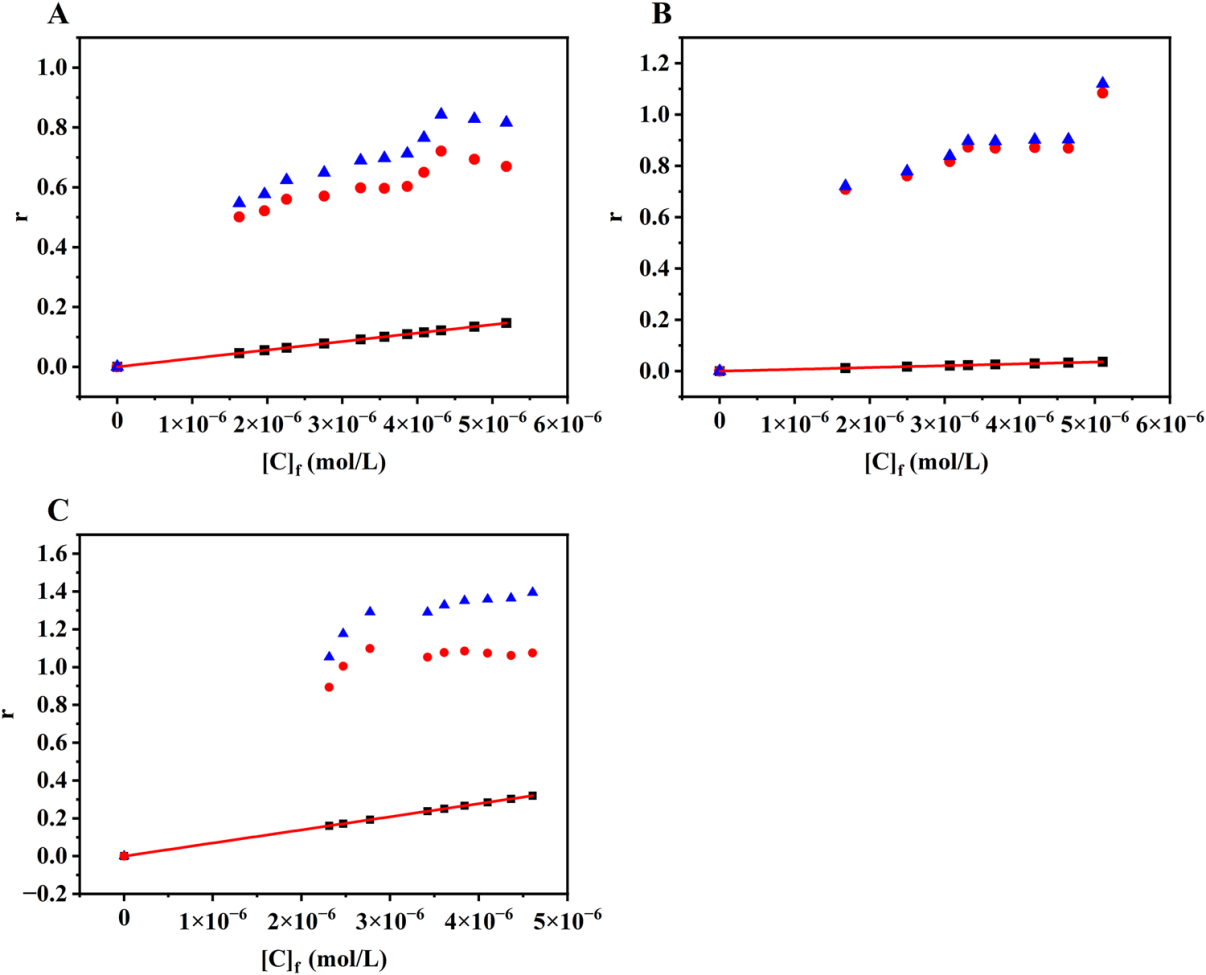
Isotherms for the binding of cyt *c* to three different aptamers based on PACE‐FA: (A) Apt40, (B) Apt61, and (C) Apt76. Triangles represent the apparent binding isotherms of the aptamers obtained directly from the experimental data; squares represent linear nonspecific binding, which is deduced from the slope of the linear part of the apparent binding curve; and circles represent specific binding obtained by subtracting the nonspecific binding component from the results of the total binding of the aptamers.

To accurately and quantitatively determine the specific binding constants corresponding to cyt *c*–aptamer complexes, Equation (4) was used to fit the total binding isotherm in Figure 5, and the corresponding results are listed in Table 1. It is noticeable from the graph that two distinct binding profiles are present for the three cyt *c* aptamers. A rough estimation can be done by measuring the slope of the linear part of the binding curve, which is dominated by nonspecific binding. The nonspecific binding factor *λ* value of Apt61 is significantly smaller than that of the other two aptamers. The binding isotherm shows subtle differentiation between total and specific binding for Apt61, shown in Figure 5A, which very likely suggested specific binding. The binding curves of Apt40 and Apt76 are estimated to have contributions from both specific binding and nonspecific binding, simultaneously. By extracting the specific binding component from the measured binding constant, the binding constant of Apt76 (1.53 × 10^6^ M^−1^) was determined to be significantly greater than those of the other two aptamer sequences. This result can be used to rationalize the earlier claim that, being the longest of the three aptamers, Apt76 adopts a multi‐loop structure and specifically binds to cyt *c*, providing a detection limit of approximately 0.50 nM based on the results obtained using thickness shear mode acoustics method [2]. By subtracting the contribution of the nonspecific binding component from the total binding constant, it was further determined that all three aptamers interacted with the target, cyt *c*, in almost 1:1 stoichiometry.

### 4.4 Determination of Binding Constants Using Fluorescence Spectroscopy

To further verify the PACE‐FA results, the interaction between cyt *c* and each of the three aptamers was analyzed using fluorescence titrations. As shown in Figure S4, as the cyt *c*:aptamer molar ratio was increased from 1:1 to 1:7, the emission signal of cyt *c* at 292 nm gradually decreased. With the addition of each aptamer to the cyt *c* solution, no characteristic wavelength shift of the cyt *c* emission maximum was observed in the fluorescence spectrum, indicating that the aptamer and its target (i.e., cyt *c*) interacted noncovalently. It has been suggested that hydrophobic interactions between cyt *c* and aptamers change the microenvironment around the cyt *c* molecules, causing a decrease in the fluorescence quantum yield, which results in a lower fluorescence intensity at 292 nm. However, additional studies are required to further characterize the binding mode of the aptamers to the target protein cyt *c*.

**FIGURE 6:**
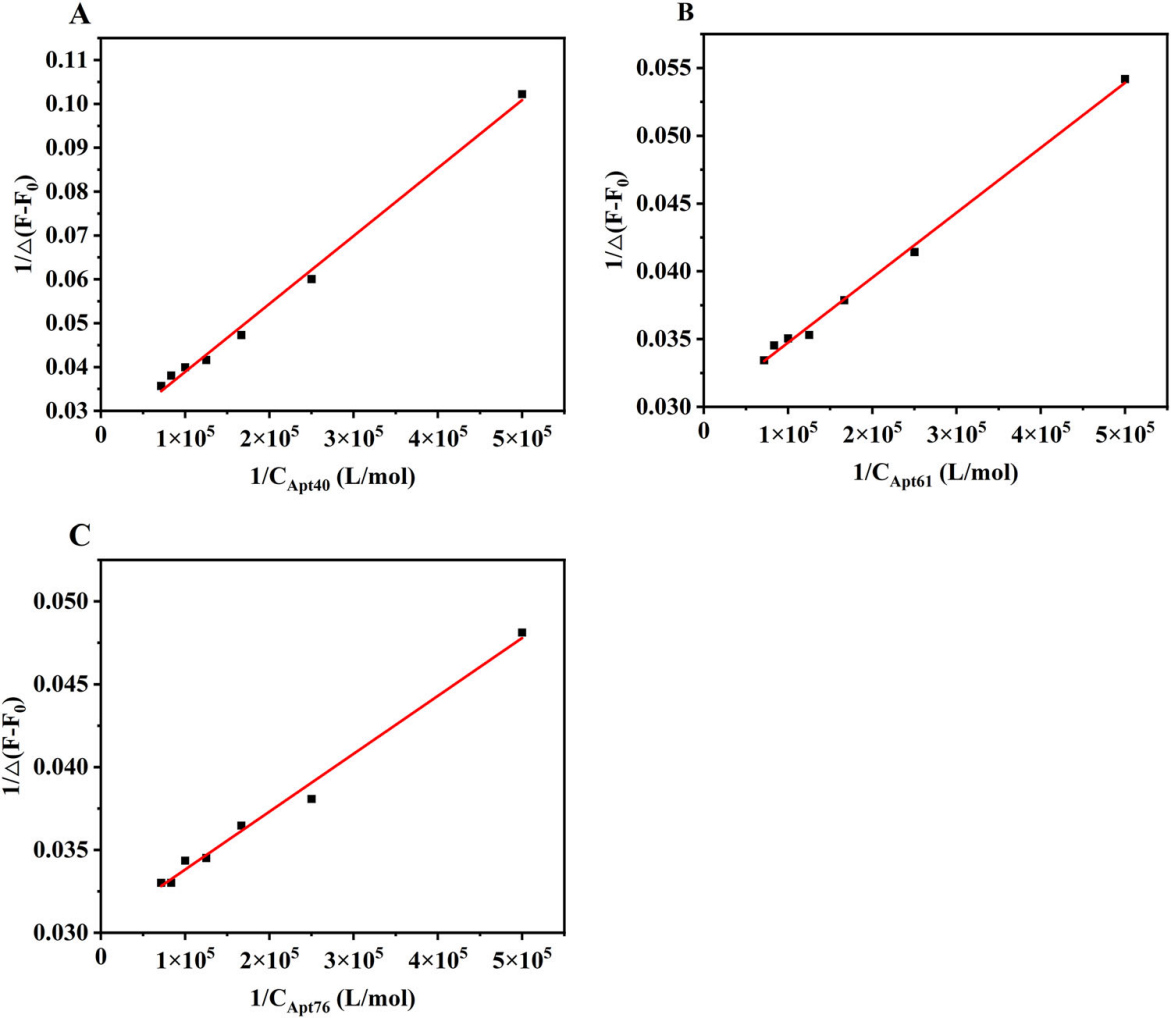
Plots of 1/(*F*_0_ − *F*) versus 1/*C*_apt_ obtained using the fluorescence titration data of cyt *c* versus its aptamer: (A) Apt40, (B) Apt61, and (C) Apt76.

According to Equation (5), we plotted 1/(*F*_0_ − *F*) versus 1/*C*_apt_. In the resulting linear plot (Figure 6), the intercept corresponds to 1/*F*_0_, whereas the slope provides 1/*K*_a_ *F*_0_. Thus, *K*_a_ could be determined from the slope of the linear fit. The fitting results for the three aptamers are presented in Table 1. For the same target protein (i.e., cyt *c*), the order of the binding ability of the three aptamers was Apt76 > Apt61 > Apt40, consistent with PACE‐FA results. Among these three cyt *c* aptamers, only Apt61 has been reported for its dissociation constant (*K*_d_ ∼ 4.6 µM) [46]. The calculated binding constant of Apt61 is in close agreement with that of previous reports. For these three cyt *c* aptamers, the longer the chain was, the larger the *K*_a_ value was, given that the same target protein was used. These results suggest that the binding constant of Apt76 was larger than those of the other two sequences, indicating a good binding affinity of Apt76 for cyt *c*. The reason for the slightly lower binding constant of Apt40 obtained by the fluorescence assay compared with that determined by PACE‐FA can be attributed to the coexistence of specific and nonspecific binding in the fluorescence assay. Thus, the binding constants obtained from the PACE‐FA method were verified through fluorescence titration experiments, demonstrating the prospects of the PACE‐FA method in studying protein–aptamer interactions.

## 5 Concluding Remarks

A CE‐based approach was developed for characterizing protein–DNA interactions, in this case, cyt *c*–aptamer interactions. PACE‐FA is proven effective for the analysis of larger biomolecules. This technique not only significantly reduced the analysis time but also maintained the level of accuracy using the mobility‐based correction method. Three cyt *c* aptamers (Apt40, Apt61, and Apt76) were used in various sensing approaches. However, there is no consensus on their affinity and specificity for cyt *c* detection. We used this reference‐free method based on the CE‐FA data to extract specific binding constants by subtracting the effect of nonspecific binding. We demonstrated this approach to processing data obtained from PACE‐FA to evaluate important binding characteristics of target–aptamer systems. Apt76 was found to exhibit good binding strength (*K*_a_: 1.53 × 10^6^ M^−1^) and specificity when interacting with the cyt *c* target, and all three aptamers interacted with cyt *c* in nearly 1:1 stoichiometry. This study demonstrates that PACE‐FA can be used as a more common tool for the analysis of biomacromolecular interactions and complex high‐order binding systems. Compared with other binding approaches, this nonperturbing analytical method improves the accuracy and specificity of the analysis.

## Conflicts of Interest

The authors declare no conflicts of interest.

## Supporting information




**Supporting File 1**: elps70018‐sup‐0001‐SupMat.docx

## Data Availability

All data included in this study are available upon request by contacting the corresponding authors.
