# Supplementary material for: Characterization of the Specific Binding Between Aptamers and Cytochrome c With Pressure‐Assisted Capillary Electrophoresis Frontal Analysis
Source: Electrophoresis. 2025 Sep 1;46(19):1461–70. doi: 10.1002/elps.70018 (PMC12572689; doi:10.1002/elps.70018)
Supplement: Supplementary file 1 — Supporting File 1: elps70018‐sup‐0001‐SupMat.docx [file ELPS-46--s001.docx]

**Supporting Information**

**Characterization of the specific binding between aptamers and cytochrome c with pressure-assisted capillary electrophoresis frontal analysis**

Shuanghao Wang,^1,3^ Chunliang Li,^1^ Shuangshuang Wang,^1^ Huihui Li,^1,2,*^ David Da Yong Chen^1,2,*^

^1^*State Key Laboratory of Microbial Technology, Jiangsu Collaborative Innovation Center of Biomedical Functional Materials, Jiangsu Key Laboratory of New Power Batteries, School of Chemistry and Materials Science, Nanjing Normal University, Nanjing 210023, China*

^2^*Department of Chemistry, University of British Columbia, Vancouver, BC, Canada V6T 1Z1*

^3^*Jiangyan High School of Jiangsu Province, Taizhou 225599, P. R. China*


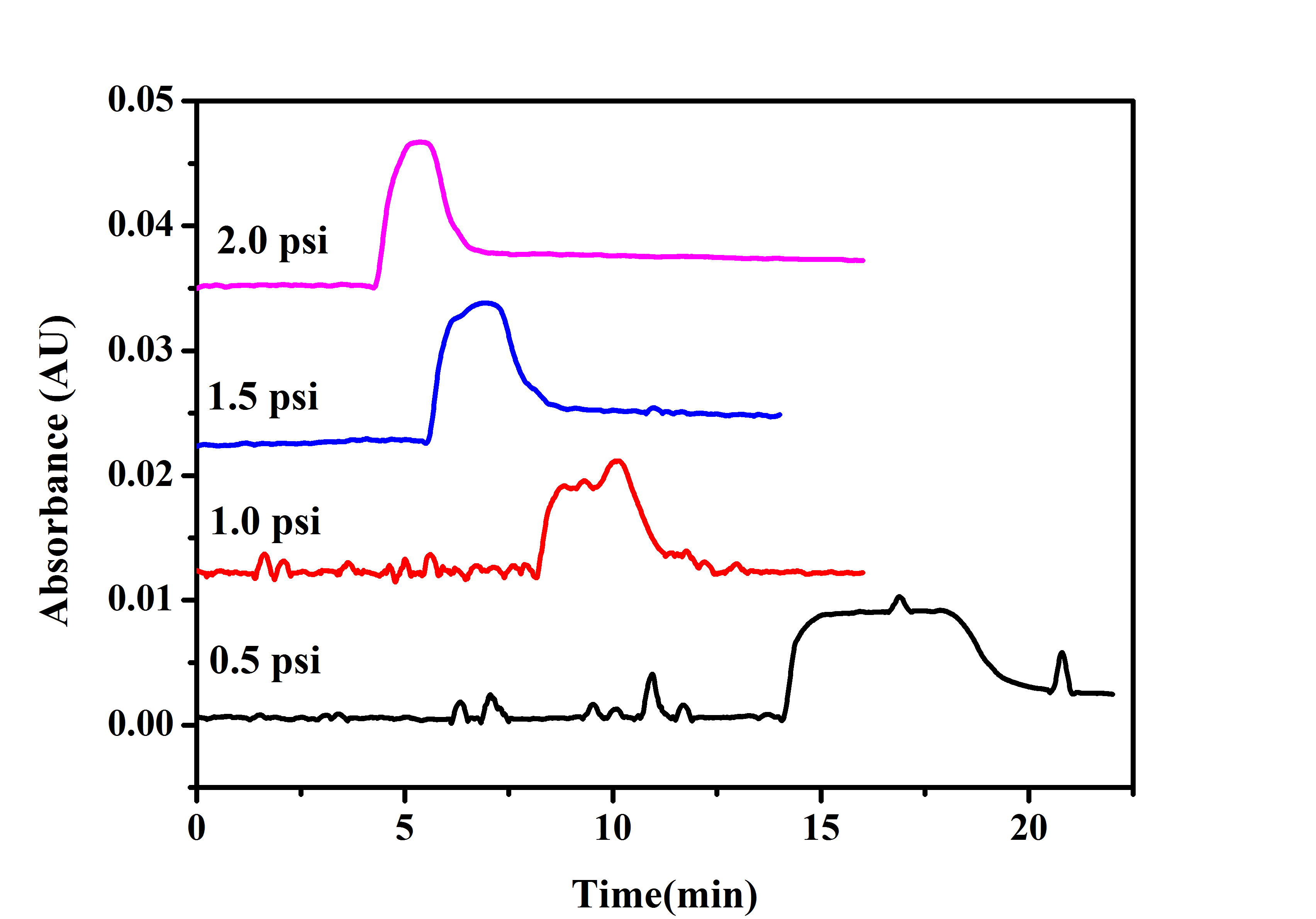


Figure S1. PACE-FA electropherograms of 8.0 μM cyt c in 10 mM NH_4_OAc at different external pressures. Injection time: 90 s, injection pressure: 1.0 psi (6.9 kPa), separation voltage: 20 kV.


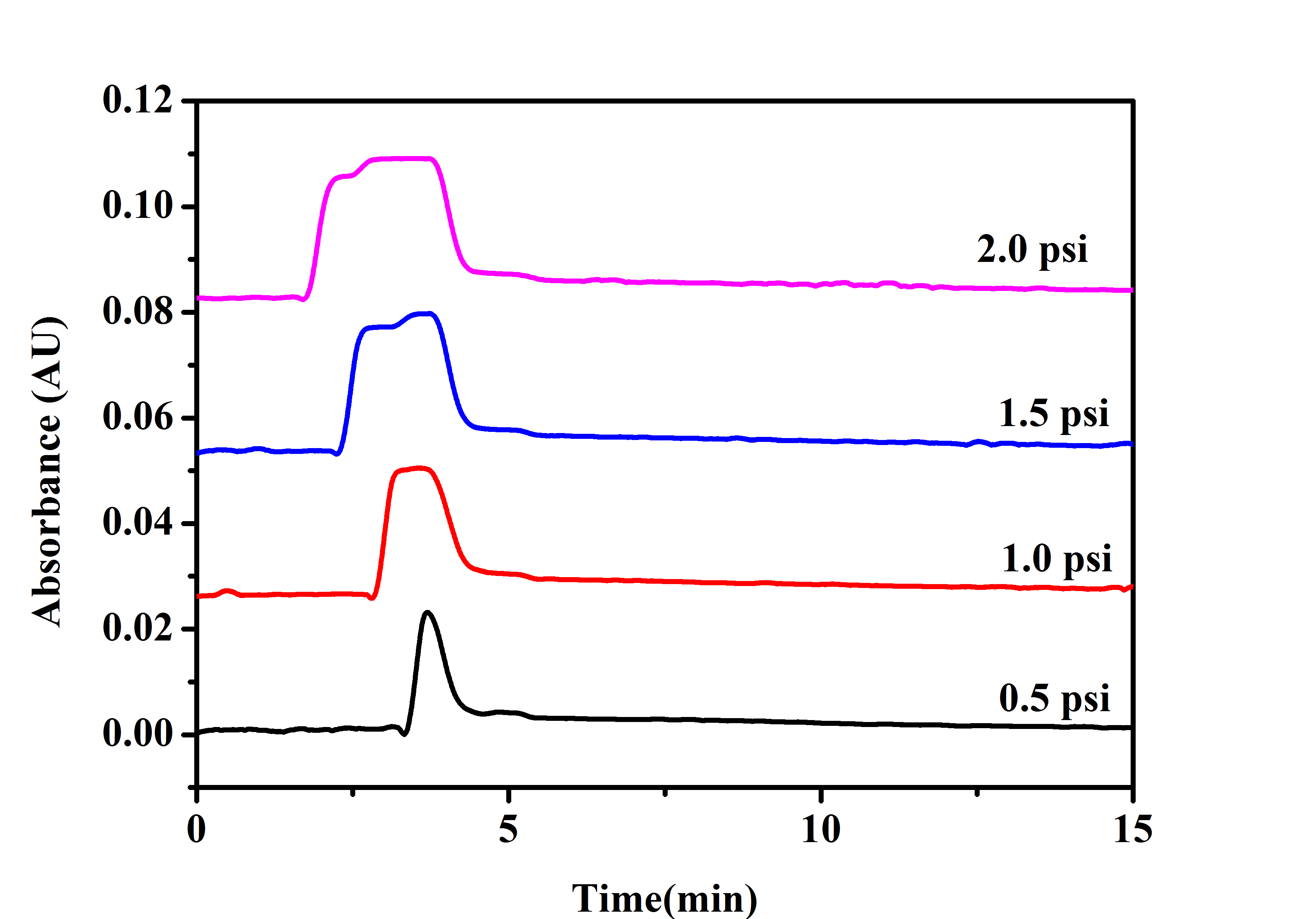


Figure S2. PACE-FA electropherograms of 8.0 μM cyt c in 10 mM NH_4_OAc at different injection pressures. Injection time: 90 s, separation voltage: 20 kV, external pressure: 2.0 psi (13.8 kPa).


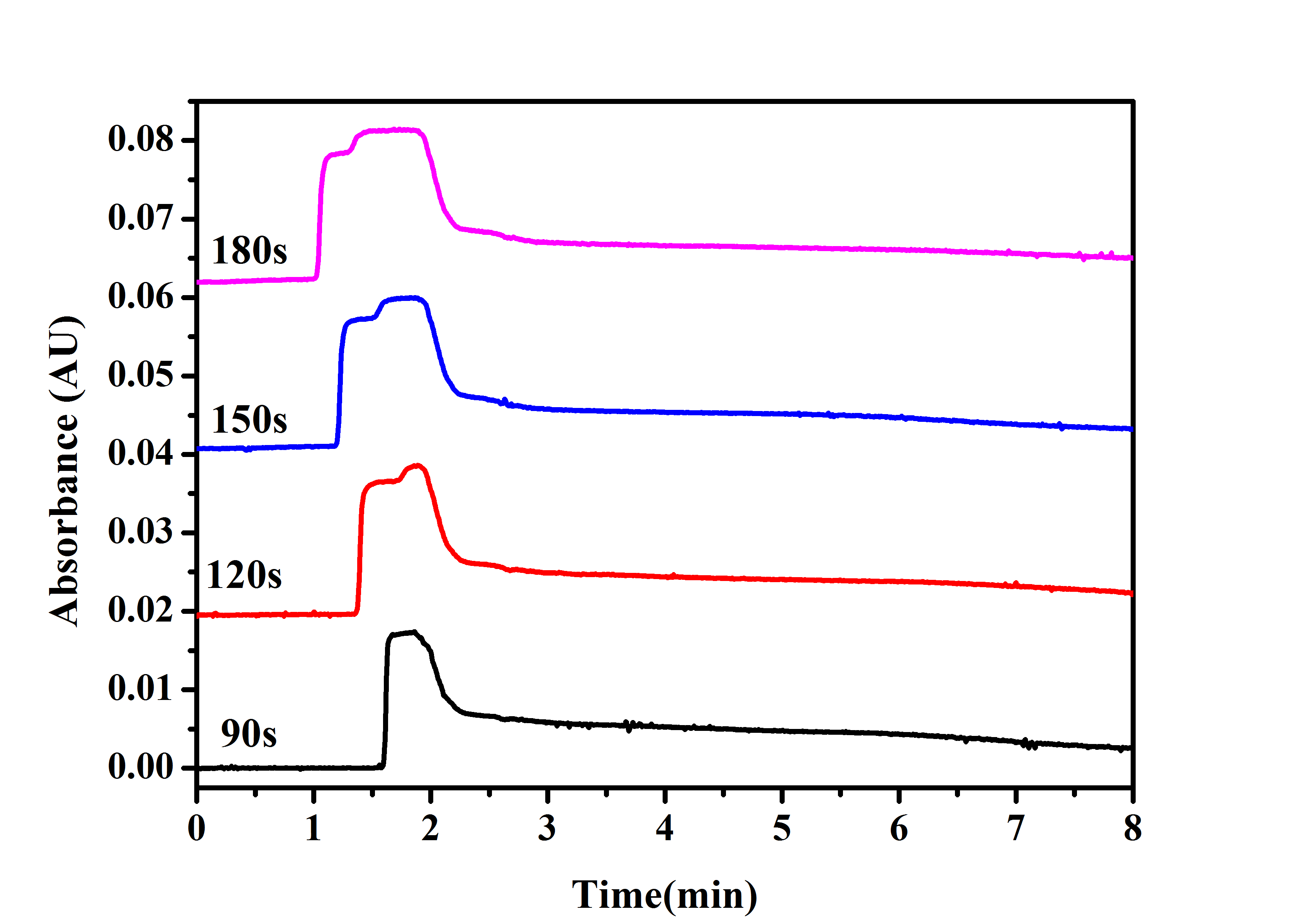


Figure S3. PACE-FA electropherograms of 8.0 μM cyt c in 10 mM NH_4_OAc at different injection time. Injection pressure: 1.0 psi (6.9 kPa), separation voltage: 20 kV, external pressure: 2.0 psi (13.8 kPa).


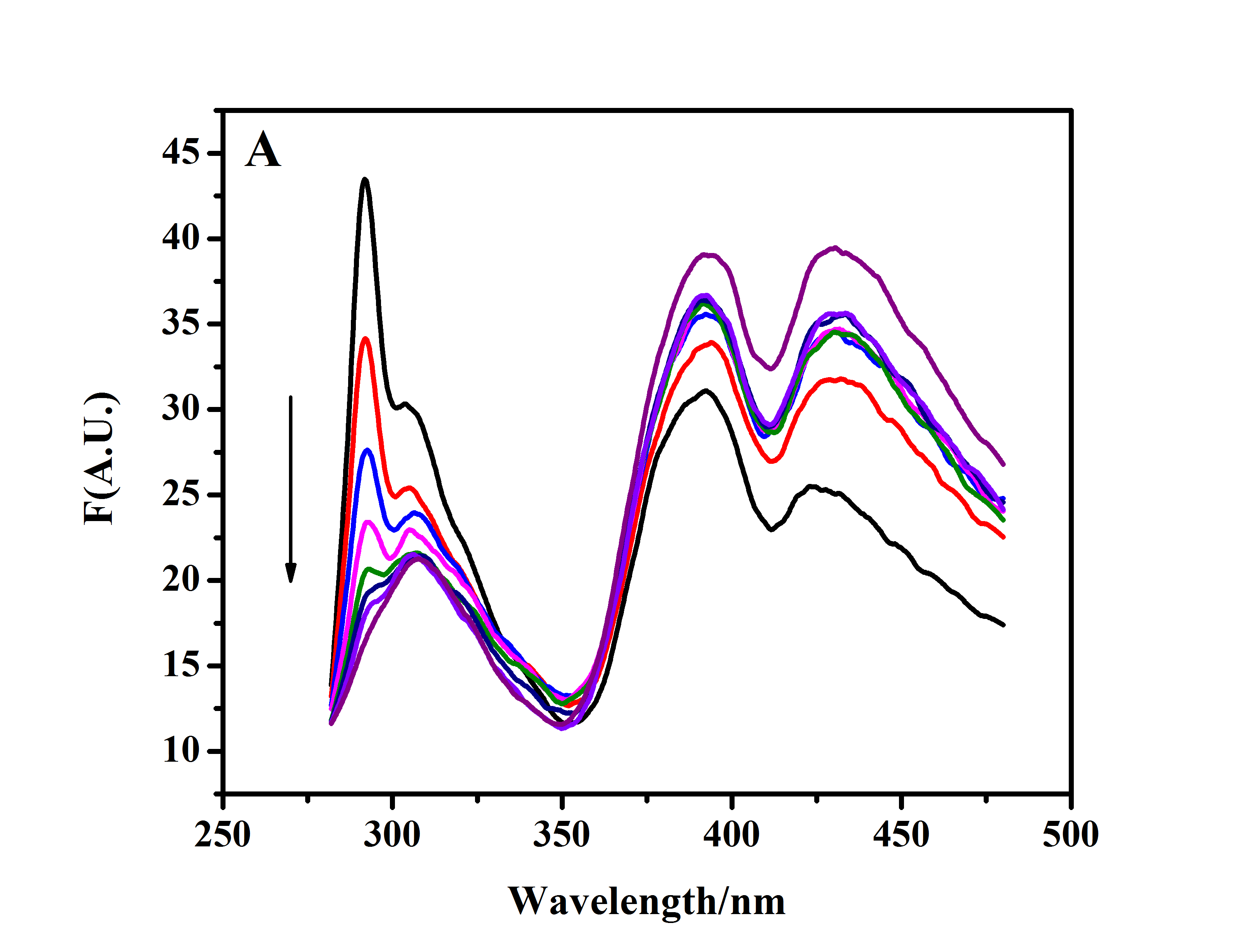

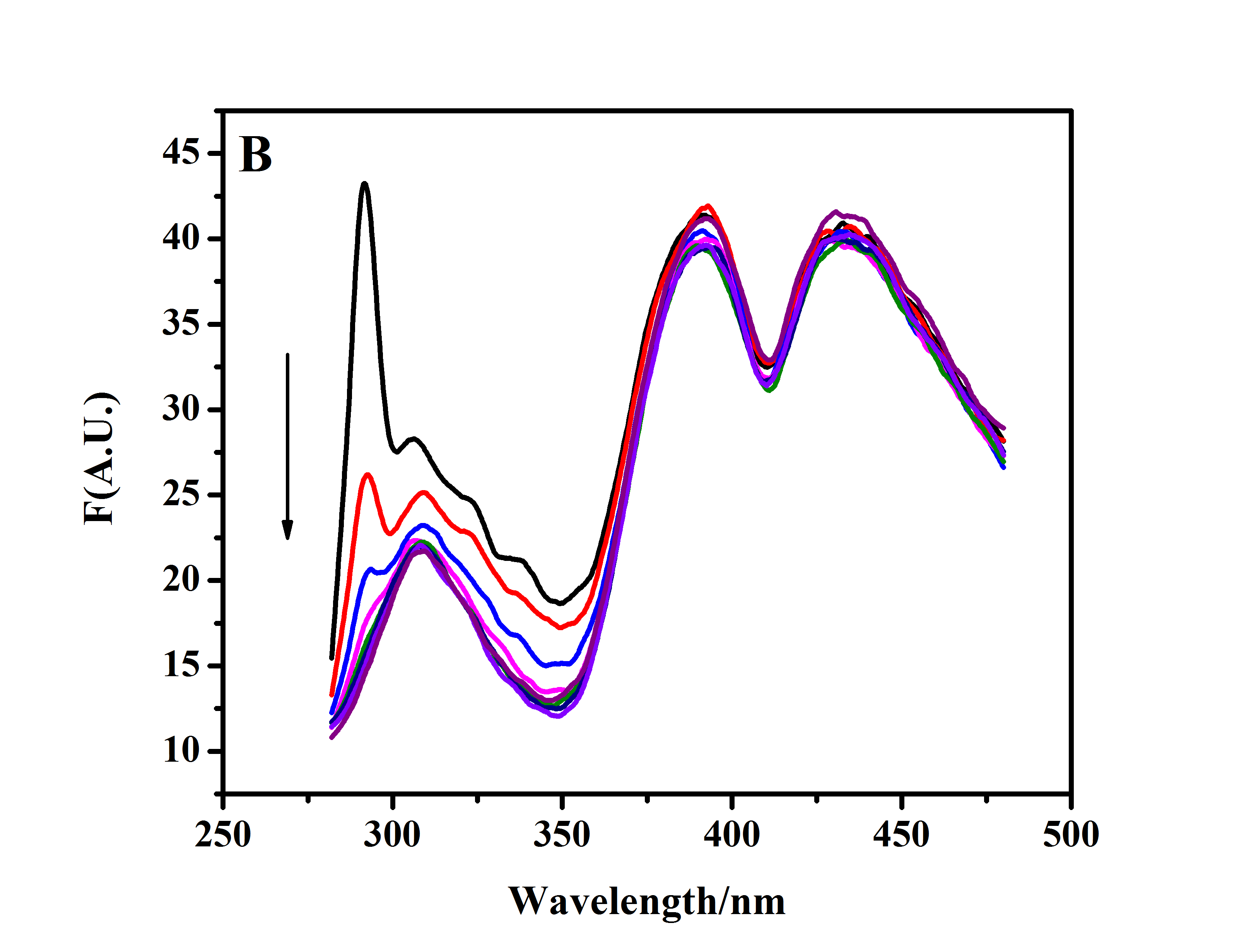

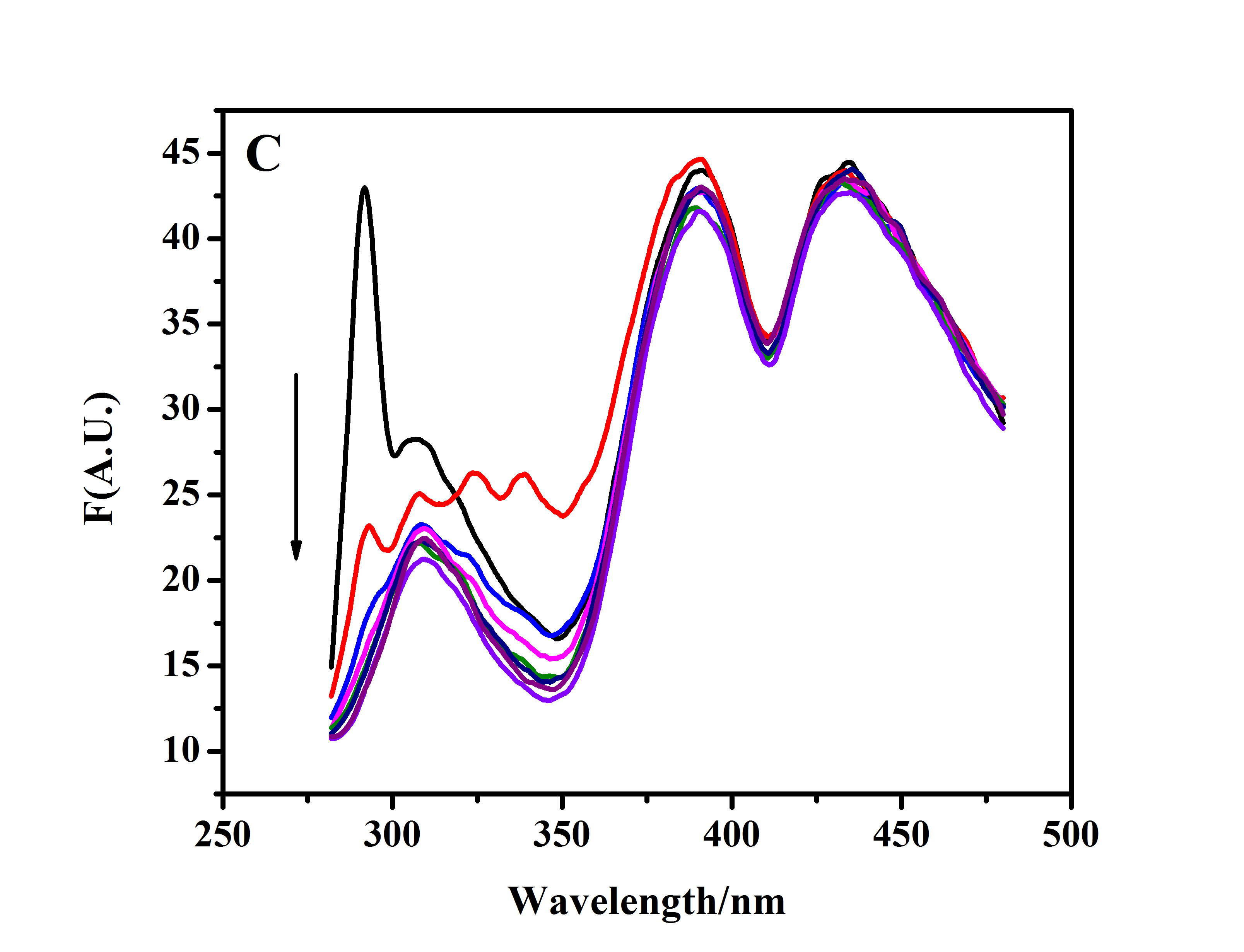


Figure S4. Fluorescence spectra of cyt c (2.0 μM) in the presence of the aptamer from 2.0 - 14.0 μM. (A) Apt40; (B) Apt61; (C) Apt76. Excitation wavelength: 262 nm.
